# Glioblastoma Recurrence Correlates With Increased APE1 and Polarization Toward an Immuno-Suppressive Microenvironment

**DOI:** 10.3389/fonc.2018.00314

**Published:** 2018-08-13

**Authors:** Amanda L. Hudson, Nicole R. Parker, Peter Khong, Jonathon F. Parkinson, Trisha Dwight, Rowan J. Ikin, Ying Zhu, Jason Chen, Helen R. Wheeler, Viive M. Howell

**Affiliations:** ^1^The Brain Cancer Group, Bill Walsh Translational Cancer Research Laboratory, Kolling Institute, St Leonards, NSW, Australia; ^2^Northern Sydney Local Health District, St Leonards, NSW, Australia; ^3^Sydney Medical School Northern, University of Sydney, Sydney, NSW, Australia; ^4^Cancer Genetics, Hormones and Cancer Group, Kolling Institute, St Leonards, NSW, Australia; ^5^Hunter New England Health, New Lambton, NSW, Australia; ^6^Department of Anatomical Pathology, Northern Sydney Local Health District, St Leonards, NSW, Australia

**Keywords:** glioblastoma, recurrence, immuno-suppression, macrophage polarization, microenvironment

## Abstract

While treatment with surgery, radiotherapy and/or chemotherapy may prolong life for patients with glioblastoma, recurrence is inevitable. What is still being discovered is how much these treatments and recurrence of disease affect the molecular profiles of these tumors and how these tumors adapt to withstand these treatment pressures. Understanding such changes will uncover pathways used by the tumor to evade destruction and will elucidate new targets for treatment development. Nineteen matched pre-treatment and post-treatment glioblastoma tumors were subjected to gene expression profiling (Fluidigm, TaqMan assays), *MGMT* promoter methylation analysis (pyrosequencing) and protein expression analysis of the DNA repair pathways, known to be involved in temozolomide resistance (immunohistochemistry). Gene expression profiling to molecularly subtype tumors revealed that 26% of recurrent post-treatment specimens did not match their primary diagnostic specimen subtype. Post-treatment specimens had molecular changes which correlated with known resistance mechanisms including increased expression of APEX1 (*p* < 0.05) and altered *MGMT* methylation status. In addition, genes associated with immune suppression, invasion and aggression (*GPNMB, CCL5, and KLRC1*) and polarization toward an M2 phenotype (*CD163* and *MSR1*) were up-regulated in post-treatment tumors, demonstrating an overall change in the tumor microenvironment favoring aggressive tumor growth and disease recurrence. This was confirmed by *in vitro* studies that determined that glioma cell migration was enhanced in the presence of M2 polarized macrophage conditioned media. Further, M2 macrophage-modulated migration was markedly enhanced in post-treatment (temozolomide resistant) glioma cells. These findings highlight the ability of glioblastomas to evade not only the toxic onslaught of therapy but also to evade the immune system suggesting that immune-altering therapies may be of value in treating this terrible disease.

## Introduction

Glioblastoma (grade IV astrocytoma) is the most common and aggressive form of brain cancer and has a very grim prognosis. Despite aggressive treatment involving maximal surgical resection, radiation and concomitant and adjuvant chemotherapy with temozolomide (TMZ), the median survival time is approximately 15 months, and the five-year survival rate remains at a staggering 3–5% (1). While these multimodal treatments may prolong life, recurrence is inevitable with tumors either being inherently resistant, as is the case in approximately 50% of glioblastomas (2) or acquiring resistance (3).

Studies investigating mechanisms of resistance have uncovered common themes including dysregulation in DNA repair enzymes, including increased levels of O^6^-methylguanine DNA methyltransferase (MGMT), and over-expression or amplification of EGFR, and loss of p53 and PTEN [reviewed in (4)]. However, such investigations have generally been performed on the initial pre-treatment tumor where only inherent resistance mechanisms would be observed. Limited data exists on how much treatment affects the molecular profiles of glioblastomas and how these tumors adapt to withstand these treatment pressures (5). Such changes may have important clinical consequences for patient management. Understanding such changes will uncover pathways used by the tumor to evade destruction and will elucidate new targets for treatment development. In addition, identifying such changes will also highlight the importance of testing recurrent samples which may have important consequences to patient management.

In this study, nineteen matched pre-treatment and post-treatment glioblastoma tumors were analyzed to identify molecular changes following treatment and recurrence of disease with a particular focus on the DNA repair pathways. We hypothesize that molecular changes will correlate with resistance mechanisms and the ability of the tumor to escape from the host's immune system.

## Methods

### Clinical characteristics

Glioblastoma samples were from patients diagnosed and treated at Royal North Shore (RNS) and North Shore Private Hospitals between August 2012 and July 2013. This study was carried out in accordance with the recommendations of the Northern Sydney Local Health District Human Research Ethics Committee. The protocol was approved by the Northern Sydney Local Health District Human Research Ethics Committee under protocols 0211-171M and 1306-212M. All subjects gave written informed consent in accordance with the Declaration of Helsinki. Tumor histology was reviewed by neuropathologist (J.C.) and a diagnosis of glioblastoma confirmed. All tumor samples, both pre-treatment and post-treatment, were classified as grade 4 glioblastomas. Clinical data collected included age at diagnosis, gender, treatment and survival, with a minimum of 15 months follow-up on all cases (Table 1).

**Table 1:** Cohort details.

**Case # (male ♂/female♀)**	MGMT promoter methylation status; M: >13%, U: <13%		**Treatment received between specimens**	**Age at diagnosis (years)**	**Time to recurrence (diagnosis to 2nd surgery, months)**	**Overall survival since glioblastoma diagnosis (months)**
	**Pre-treatment specimen (a)**	**Post-treatment specimen (b)**				
4♂	U	U	Stupp	53	6	18
5♂	U	U	Stupp	63	35	50
10♂	U	U	Stupp	81	7	13
19♀	U	U	Stupp	57	14	15
20♂	U	U	Stupp	60	7	17
25♂	M	M	Stupp	60	7	9
29♂	U	U	Stupp	40	29	47
33♀	U	U	Stupp	64	11	18
38♂	U	U	Stupp	54	3	15
40♀	U	U	Stupp	48	3	10
42♀	U	U	Stupp	64	7	19
23♀	U	U	RT-adjuvant TMZ	76	4	10
30♂	M	M	RT-adjuvant TMZ	64	3	3
35♂	U	U	RT-adjuvant TMZ	78	10	12
26♂	M (13.75)	U (2.0)	RT-TMZ Procarb	35	31	40
7♂	M (15.75)	U (2.0)	RT-Procarb TMZ Cilengtide	44	13	22
13♀	U	U	RT-Procarb TMZ Cilengtide	58	3	12
16♂	U	U	RT-Procarb TMZ Cilengtide	69	4	15
32♀	U	U	RT-Procarb TMZ Cilengtide	41	13	33
			**Mean**	**58.4**	**11.1**	**19.9**
			**Median**	**60**	**7**	**15**

*M, Methylated promoter; U, Unmethylated promoter; RT, radiotherapy; TMZ, temozolomide chemotherapy; Stupp, concurrent RT+TMZ followed by adjuvant TMZ. For cases where variability between specimens was detected; the percentage methylation is shown in brackets*.

### Fresh frozen tumor tissue

A cohort of 19 matched pre-treatment and post-treatment glioblastoma tumor specimens were available. During surgery, tumor specimens were taken and immediately snap frozen and stored at −80°C for further analysis.

### Extraction of DNA and RNA

DNA and RNA were extracted using the Allprep DNA/RNA/Protein kit (Qiagen, Valencia, CA) as per manufacturer's instructions. For all samples, purity was assessed using a Nanodrop ND-1000 (Thermo Scientific, Wilmington, DE) and for sequencing, DNA samples were further quantified using the Qubit dsDNA HS Assay Kit (Q32851, Life Technologies, Mulgrave, Victoria), performed on the Qubit Fluorometer 1.0 (Life Technologies).

### Analysis of *IDH1* mutation status

*IDH1* mutation status was determined by Sanger sequencing of exon 4. DNA was amplified by polymerase chain reaction (PCR), using primers spanning exon 4 (5′-CATTT GTCTG AAAAA CTTTG CTT-3′ (forward) and 5′-TCACA TTATT GCCAA CATGAC-3′ (reverse); amplicon size: 359 bp). PCR products were purified using the DNA Clean and Concentrator Kit (Zymo Research, Irvine, CA), and commercially sequenced (Australian Genome Research Facility, Westmead, Australia).

### Determination of *MGMT* promoter methylation status

DNA extracted from frozen tumor tissue was tested for *MGMT* promoter methylation by commercial pyrosequencing (University of Sydney). The assay threshold was determined by averaging the percent methylation at 4 sites in exon 1 of the human *MGMT* gene (Chr 10: 131,265,519-131,265,537) in 4 non-neoplastic brain tissue samples (previously confirmed as being unmethylated by both pyrosequencing and methylation-specific PCR), and applying 2 standard deviations as previously reported by Dunn et al (6). Control samples were analyzed in 3 to 5 independent pyrosequencing runs, giving a mean of 5.49% (SD 3.85) and a positive methylation assay threshold of 13% (SI Table 1 and 2).

### Gene expression analysis

Following quality analysis and quantification, 1 μg RNA was treated with DNase 1, amplification grade (Life Technologies) and reverse transcribed using the SuperScript III First Strand Synthesis SuperMix Kit (Life Technologies) according to the manufacturer's instructions. Gene expression was then analyzed commercially using the Fluidigm 96.96 BioMark HD System (Ramaciotti Gene Analysis Centre, Randwick, NSW, Australia) or in-house using the Applied Biosystems 7900HT real time PCR as per manufacturer's instructions. Taqman assays and identification numbers are listed in SI Table 3. The NormFinder algorithm (7) was used to compare 5 endogenous control genes included on the array (*TBP, ACTB, GAPDH, IPO8*, and *SDHA*), identifying *TBP* as being the most stable control gene. Relative expression of target genes was determined using the 2^−delta−deltaCt^ method (Fluidigm Real-time PCR analysis software), normalizing expression to *TBP* and a commercial pooled normal brain control sample (calibrator; Ambion, Life Technologies).

### Classification of transcriptional subtypes

Ct values were imported into the HTqPCR package in Bioconductor (8) and unreliable data filtered out by applying a Ct cut off value of 40. Genes with errors detected in <1% of samples were retained in the analysis by imputing median values for those samples. Samples with Ct values greater than 40 for *CCND2* were given a Ct = 40 to retain these in the analysis. *TBP* was used for delta Ct normalization. The Euclidean distance metric was used for hierarchical clustering of samples into transcriptional subclasses using a 30–gene panel previously published (9).

### *In silico* gene analysis

Kaplan Meier survival curves were generated for genes found to be significantly different in matched pre-treatment and post-treatment tumor specimens using the REMBRANDT repository from Project Betastatis. The “all tumor” sample group was used for analysis. Low (<median) or high (≥median) gene expression was used to divide the data set for survival. Correlations between genes were assessed using the TCGA GBM dataset and two gene scatterplots available on Project Betastatis (10, 11).

### Histological analysis

Histopathological analysis was performed to assess tumor quality. A small section of each tumor (3–5 mm^3^) was cut, fixed in formalin, and stained with haematoxylin and eosin (H&E) for scoring by neuropathologist (J.C.). Slides were scored for percentage volume of tumor, necrosis, and non-tumor tissue with a minimum cut off of 50% tumor required for further analysis.

### Immunohistological analysis

Immunohistochemistry was performed using 4 μm sections of pre- and post-treatment tumor specimens. All antibody details and optimized conditions are listed in SI table 4. Antigen retrieval was performed using a DAKO “Pascal” pressure cooker (121°C for 30 s, then cooled to 90°C for 10 s) except for MSH6 where universal decloaker solution was used and a temperature of 97°C for 50 min. An endogenous peroxidase block using 0.3% H_2_O_2_ for 5 min was then performed. Primary antibody incubation was followed by Mouse or Rabbit Envision (Agilent Technologies, Santa Clara, CA) for 30 min. DAB (3,3′-diaminobenzidine; Agilent Technologies) or ImmPACT™ NovaRED™ (Vector Laboratories, Burlingame, CA) was used for detection according to the manufacturer's recommendations. Positive and negative control tissues and isotype reagent controls were included.

For base excision repair (BER; *APE1*, and *PARP1*) and mismatch repair (MMR; *MSH2, MSH6, MLH1*, and *PMS2*) antibodies, for each slide up to five zones of tumor cells were marked by a neuropathologist (J.C.) and scored in a blinded fashion, scoring 100 tumor cells per zone. For BER targets, both stain intensity (scored as negative (0), weak (1), moderate (2) and strong (3)) and the percentage of positive tumor nuclei (0–25% (1); >25– <50% (2); 50–75% (3), and >75-100% (4)) were evaluated, giving an overall expression score out of 12. For MMR targets, cases with nuclei staining positively >20% up to 90% were scored 3, cases with positive cytoplasmic staining and weak/negative nuclei staining were scored 2 and cases with negative tumor nuclei and cytoplasm were scored as 1.

### Cell lines

The mouse glioma cell line GL261 was obtained from the National Cancer Institute (12) and the mouse macrophage cell line RAW 264.7 was purchased from the American Type Culture Collection (ATCC; Manassas, Virginia) (13). Pre-treatment and post-treatment GL261 cells were established by harvesting tumor cells from GL261 tumor bearing C576Bl/6 mice either not treated (treatment naïve/pre-treatment) or treated (Rx) for 3 weeks with 5 mg/kg TMZ (TMZ treated/post-treatment). These TMZ treated/post treatment cells have a 4-fold higher IC50 value then the treatment naïve/pre-treatment cells using standard MTS cytotoxicity assays (123 μM v 498 μM respectively; *p* < 0.01; SI Figure 1). All cells were cultured and maintained in DMEM media supplemented with 10% fetal bovine serum and grown in standard conditions (37°C humidified incubator with 5% CO_2_).

### Conditioned media from polarized RAW 264.7 cells

RAW 264.7 cells were harvested (by cell scraper) and seeded at 25 × 10^5^ cells/T25 flask. After 24h, cells were polarized in 5 ml serum free DMEM as follows: For M0 polarization, serum free media (SFM) alone was added. For M1 polarization, 20 ng/ml interferon gamma + 100ng/ml lipopolysaccharide (Stemcell Technologies, Vancouver) was added and for M2 polarization 20 ng/ml of IL-4, and 20 ng/ml IL-10 (Stemcell Technologies) was added. Cells were polarized for 24 h, thoroughly washed with PBS then 5 ml of fresh SFM was added. After another 24 hrs, the conditioned media (CM) was filtered (0.22 μm filter) and stored at −20°C until needed.

### Transwell migration assay

Migration assays were performed in 6.5 mm transwell plates with 8 μm pore inserts in duplicate. CM (500 μL) was added to the bottom chamber as the stimulant and 10 × 10^4^ cells in 350 μL of SFM were seeded into the upper chamber. After incubating for 24 h, the transwells were fixed in ethanol and unmigrated cells removed from the top of the membranes using cotton swabs. Membranes were then mounted onto slides using Prolong gold antifade mountant with DAPI (Life Technologies) and images taken at 200x magnification using a florescent microscope. The number of cells in 10 randomly chosen fields of view (FOV) was calculated using ImageJ.

## Statistical analysis

The statistical analysis package GraphPad Prism (v7) was used to perform *t*-tests, one-way Anova tests or log-rank (Mantel-Cox) Gehan-Breslow-Wilcoxon survival tests, with a *p*-value of < 0.05 indicating significance.

## Results

### *MGMT* promoter methylation status changed in 10% of cases following treatment and recurrence of disease

*MGMT* promoter methylation status showed that 21% of primary tumors (4/19) had a methylated promoter and this did not correlate with response to treatment or overall survival **(Table 1)**. Of note, the *MGMT* status of 2/19 patients (10%) changed from methylated to unmethylated with > 6-fold reduction in percentage methylation following treatment and recurrence of disease, similar to other reported studies (14–17).

### *APE1* protein expression increased significantly following treatment and recurrence of disease

**Figure 1:**
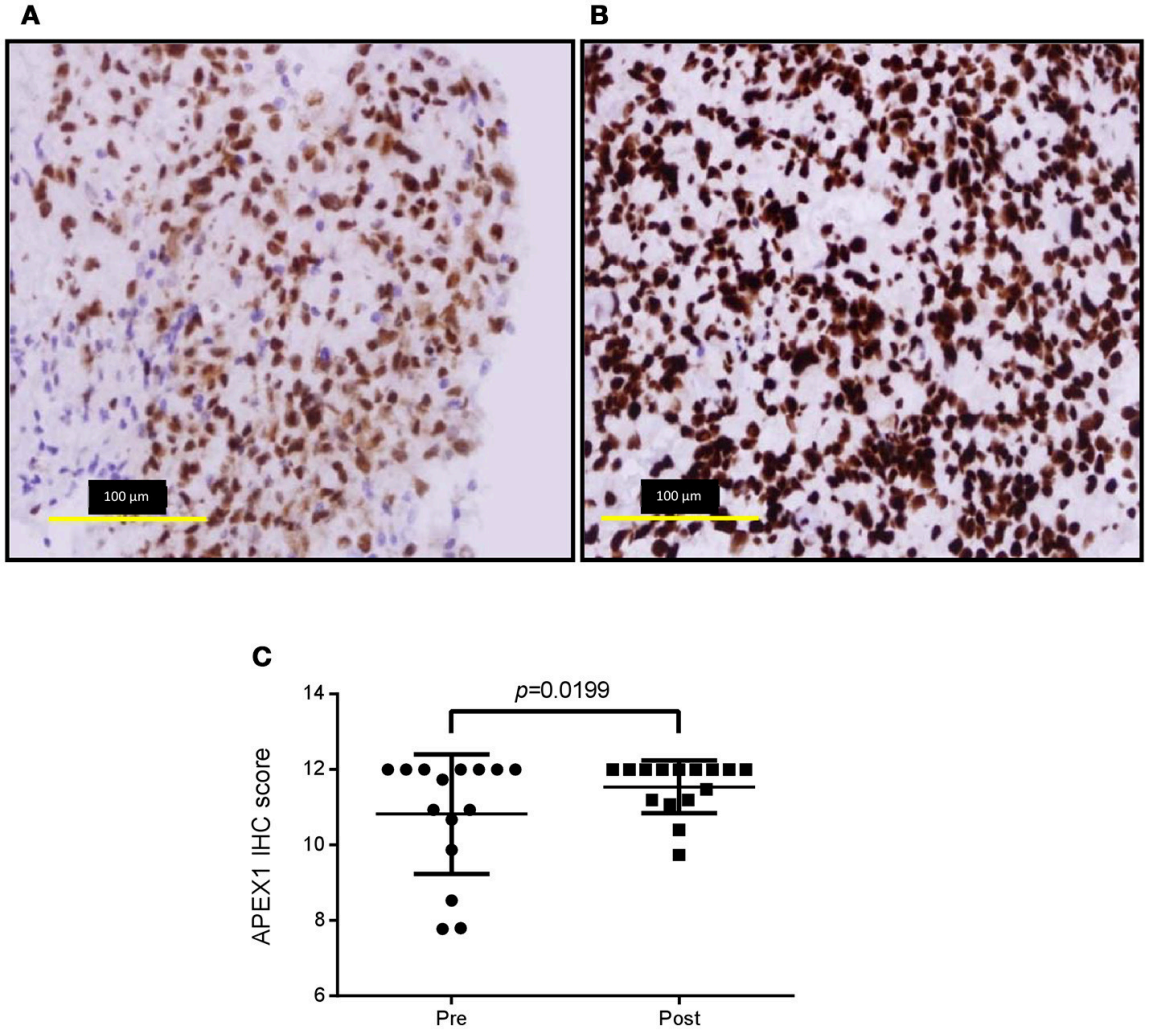
APE1 labeling of pre-treatment and post-treatment tumor specimens. Representative immunohistochemistry images of pre-treatment (**(A)**, average score 10.8 ± 1.58) and post-treatment (**(B)**, average score 11.5 ± 0.70) APE1 stained tumor sections and over-all staining scores for the cohort **(C)**. Positive and negative control tissues and isotype reagent controls were included. Staining was scored for both intensity (0-3) and percentage of positive tumor nuclei (1, 0–25%; 2, >25 – <50%; 3, 50–75%; 4, >75–100%).

The BER and MMR pathways are known to play a role in the detoxification of TMZ. As such, we investigated whether treatment and recurrence of disease correlated with changes in these pathways. Gene expression analysis of the major genes in these pathways (*APE1* and *PARP1* [BER] and *MSH2, MSH6, MLH1*, and *PMS2* [MMR]) revealed no significant differences (SI Figure 2). Targeted next generation sequencing of these genes also revealed no significant increase in the number of DNA sequence variants following treatment and disease recurrence (SI Figure 3). However, protein expression analysis did show a significant increase in APE1 following treatment and recurrence of disease (*p* < 0.05; Figure 1), a known resistance mechanism to alkylating agents and radiotherapy (18–20).

### The molecular subtype changed in 26% of cases following treatment and recurrence of disease

**Figure 2:**
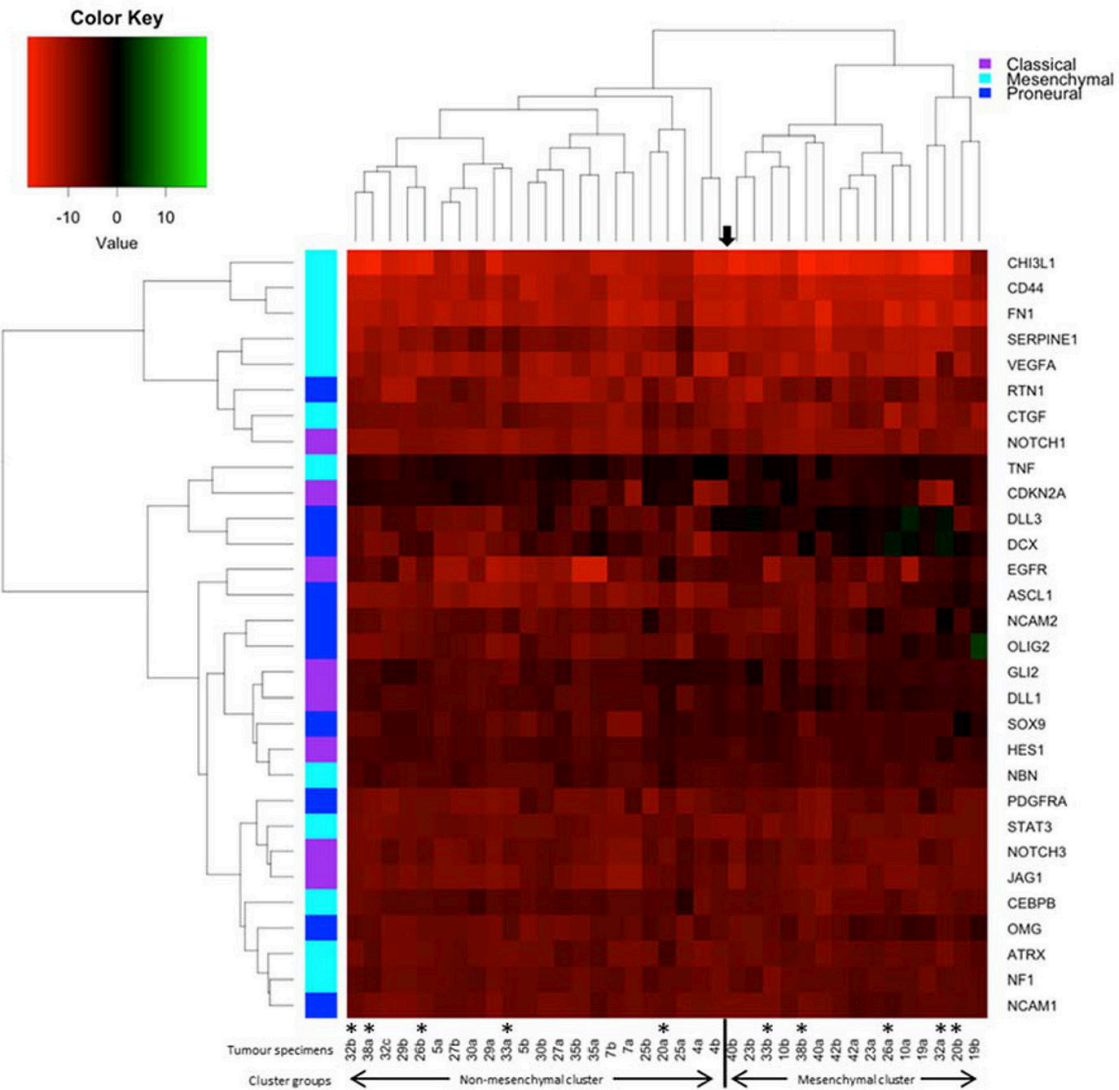
Heatmap and dendrogram of hierarchical clustering of the 38 specimens in our cohort using the 30-gene panel. Tumor specimens were clustered by Euclidean distance using qPCR results and key genes used to distinguish transcriptional subtypes. The asterisks denotes paired specimens that did not cluster together into the same molecular subtype following treatment and recurrence of disease.

A 96-gene panel was used to molecularly profile pre- and post- treatment tumors. Genes were chosen based on previous studies as classifiers for major subtypes (proneural, mesenchymal, and classical), as well as genes involved in treatment resistance. Using a previously published method (9), hierarchical clustering was able to classify the tumors into 2 distinct groups. The highest mean expression of *CHI3L1, CD44, SERPINE1*, and *CTGF* was found in one cluster identifying the tumors as mesenchymal. The 2nd cluster was unable to be classified by key genes belonging to one particular subtype having mixed expression levels of proneural (*DLL3, OLIG2, ASCL1*, and *PDGFR*) and classical (*EGFR, NOTCH3, JAG1*, and *GLI2*) genes. We identified changing molecular profiles as a result of treatment and recurrence with 5/19 (26%) primary tumors not matching their post-treatment specimen (Figure 2). In 3 of these cases, recurrence was associated with a change to a mesenchymal subtype and in one case there was a change in both subtype and *MGMT* methylation status (case #26).

### An immunosuppressive microenvironment identified in post-treatment tumors

**Figure 3:**
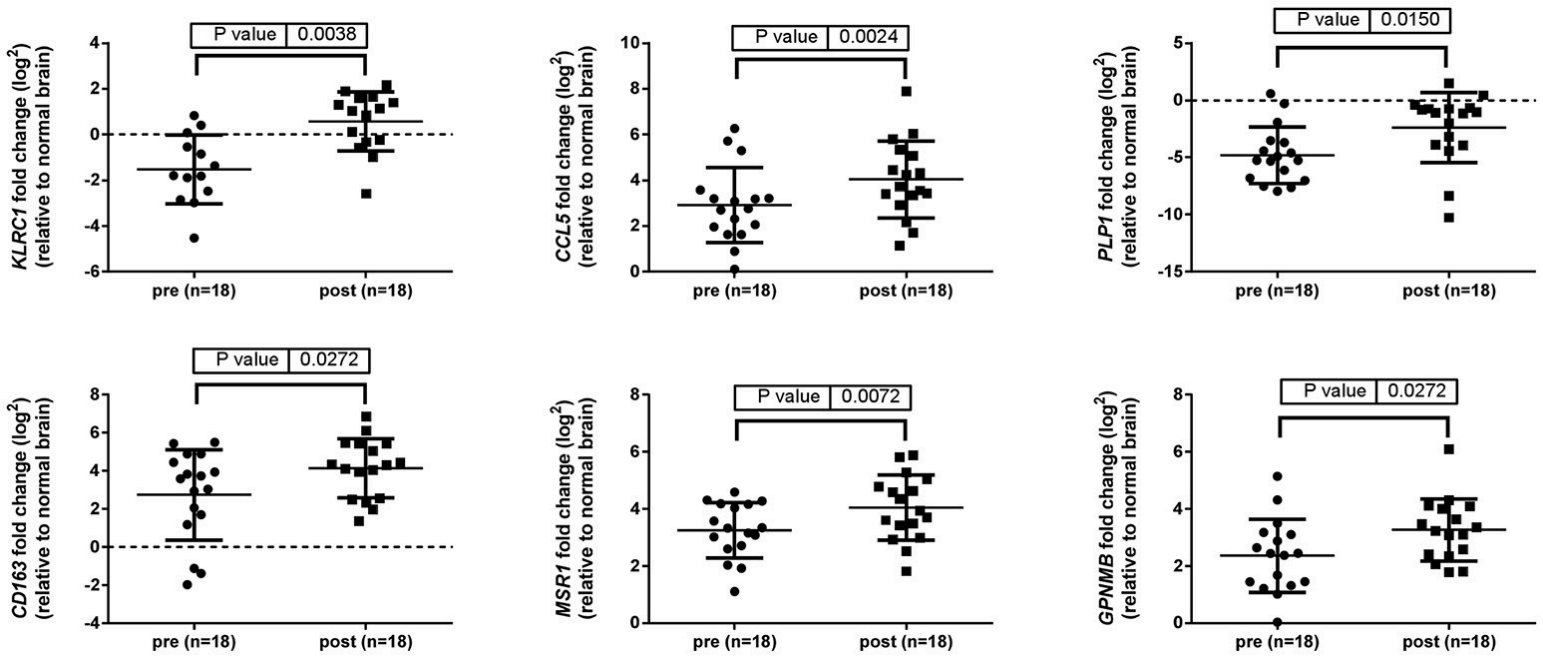
Immune related genes differentially expressed between pre-treatment and post-treatment samples. Gene expression of 96 genes was examined in tumor specimens pre- and post-treatment relative to normal brain tissue (indicated by the broken line). Results generated using TaqMan assays and normalized to *TBP*.

**Figure 4:**
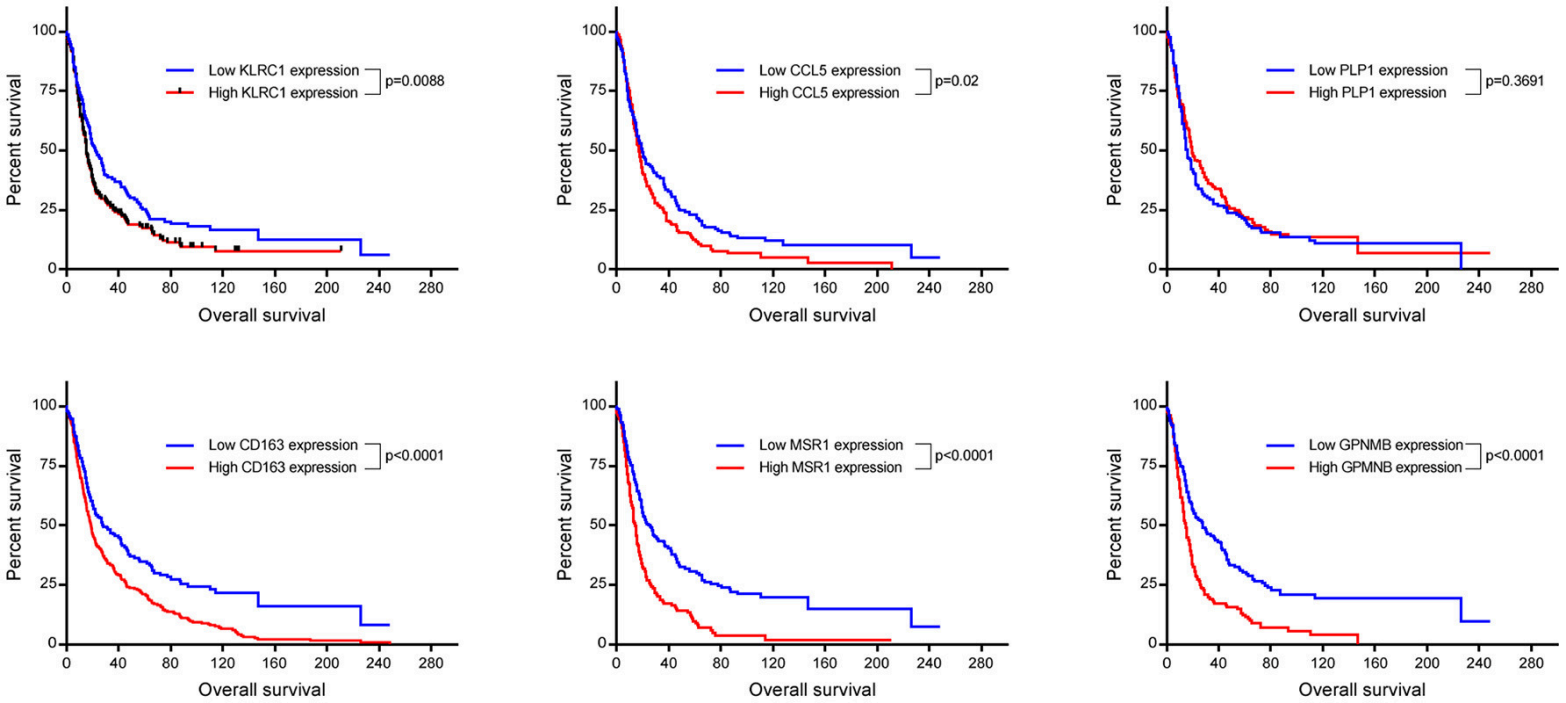
Kaplan Meyer survival curves of genes found to be significantly differentially expressed in our cohort. Data was retrieved from Project Betestatis using the REMBRANDT repository (*n* = 329 with 54 censored events).

**Figure 5:**
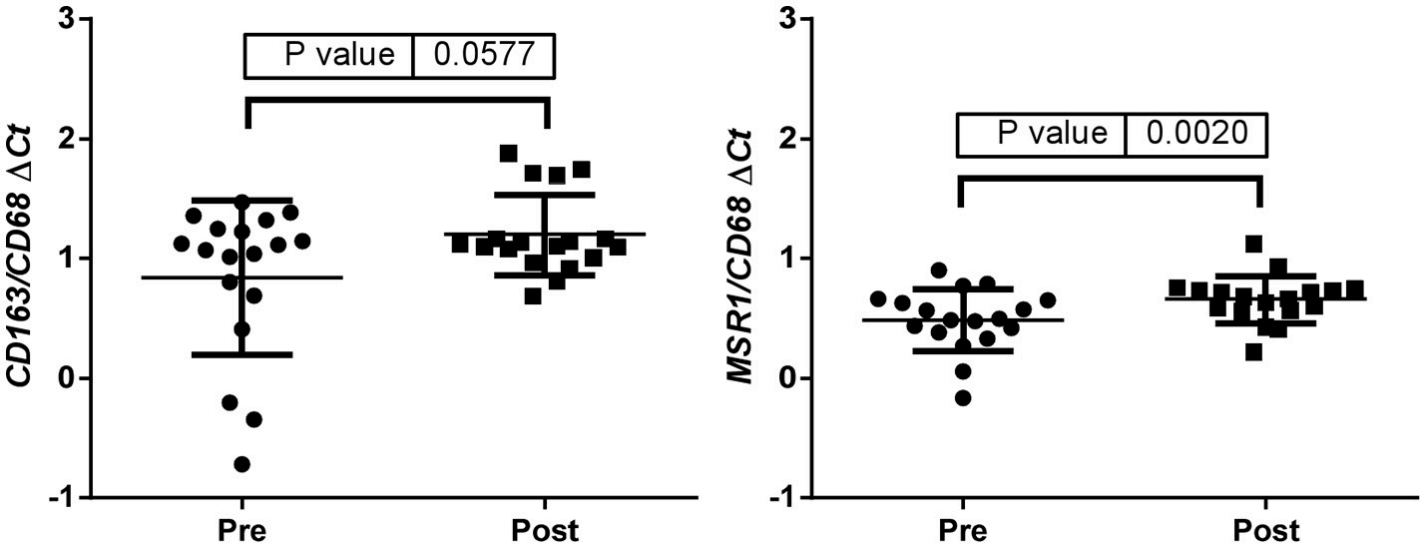
ΔCt values were used to calculate the CD163/CD68 and the MSR1/CD68 ratios indicative of a change in polarization of microglia/macrophages from an M1 (pro-inflammatory) phenotype to an M2 (anti-inflammatory and immune suppressive) phenotype in post treatment samples.

Up-regulation of a number of immune-related genes was also identified in post-treatment tumor specimens (Figure 3). Increased expression of *KLRC1, CCL5, PLP1, CD163, MSR1*, and *GPNMB* was found following treatment and tumor recurrence. To delve further into the importance of these genes, correlated gene expression and survival data was extracted from the REMBRANDT repository (10, 11). While these data include all glioma subtypes rather than matched pre-treatment and post-treatment samples, the predictive significance of these genes is evident with 6 of the 7 genes showing expression levels significantly correlated with survival (Figure 4). In addition to this, moderate to strong correlation coefficients were identified between *CD163, MSR1*, and *GPNMB* (SI Figure 4). As M2 macrophage markers, the up-regulation of *CD163* and *MSR1* in post-treatment tumors highlights a shift in macrophage polarization. To assess this further, the ratios of *CD68*, a generic macrophage marker, to *CD163* and *MSR1* were analyzed. Figure 5 shows increased M2 macrophage markers in post-treatment tumor samples indicative of a change in polarization of microglia/macrophages from an M1 (pro-inflammatory) phenotype to an M2 (anti-inflammatory and immune suppressive) phenotype in post-treatment samples.

### *In vitro* migration increased in post-treatment glioma cells (TMZ resistant) and was greatly enhanced by M2 macrophages

**Figure 6:**
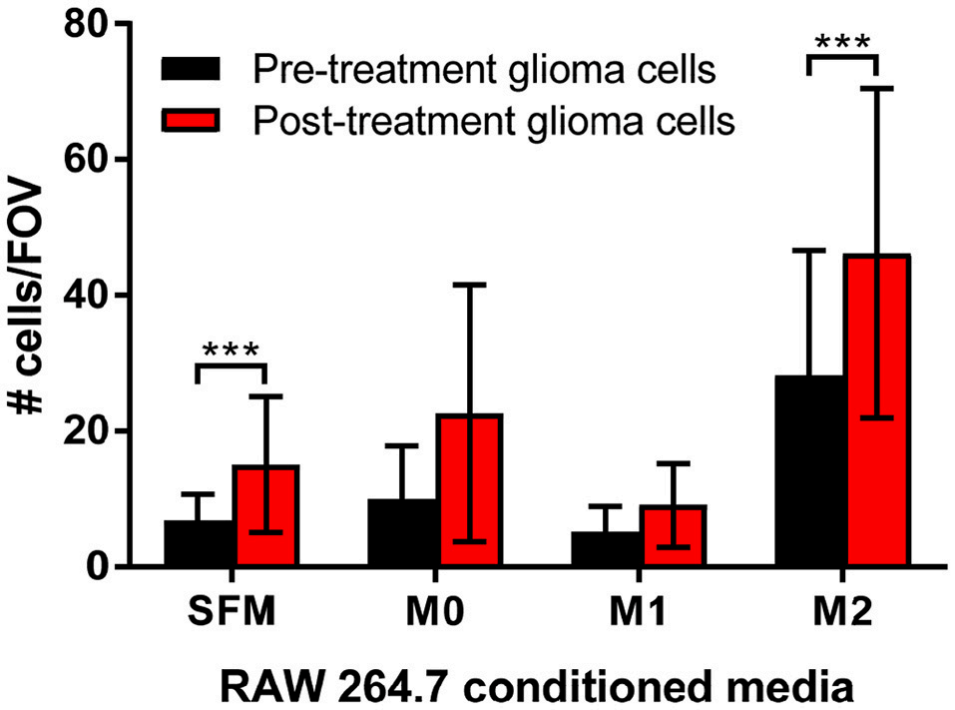
Transwell migration assays support a role for M2 macrophages in increased tumor cell migration. Matched pre-treatment/naïve glioma cells (black bars) and post-treatment/treatment resistant glioma cells (red bars) were plated into transwells and left to migrate towards control (serum free media, SFM) or conditioned media from polarized macrophages (M0, polarized with serum free media alone; M1, polarized with IFNγ/LPS; M2, polarized with IL-4/IL-10). The number of migrated glioma cells per 10 fields of view (FOV) was counted, graphed and analyzed for 3 independent experiments. The data shown is the mean ± SD of all 3 experiments combined. ***p < 0.001.

To determine the functional significance of the presence of M2 macrophages to glioma cells, transwell migration assays were performed. Matched pre-treatment (treatment naïve) and post-treatment (TMZ resistant) glioma cells were assessed for their ability to migrate toward conditioned media from polarized macrophages. Figure 6 demonstrates that post-treatment glioma cells are more migratory than their pre-treatment counterparts and, that this increased migratory potential is significantly enhanced by chemotactic molecules released from M2 macrophages.

## Discussion

Understanding how glioblastomas change over time and in response to treatment will lay the foundations for developing better treatments for this aggressive cancer. In order to undertake such a study, matched specimens taken prior to treatment and following recurrence are required. For this study we collected and characterized 19 cases of glioblastoma with matched pre- and post-treatment tumors. We identified molecular changes following treatment and disease recurrence which correlate with treatment resistance, immune suppression and a more aggressive phenotype.

Dysregulation of DNA repair pathways are known resistance mechanisms used by tumors to allow them to withstand the assault of both radiotherapy and TMZ chemotherapy. *MGMT* promoter methylation status is used clinically as a predictive marker for TMZ response (21) with increased MGMT levels (i.e., decreased methylation or unmethylated status) associated with TMZ resistance (22–24). Like other studies, we observed pre-treatment methylated tumors becoming unmethylated post-treatment, suggestive of acquiring resistance mechanisms (14–17). It should be noted however that neither *MGMT* promotor methylation status or MGMT protein expression are necessarily predictive of its repair capabilities (17). In addition, up-regulation of the BER protein APE1 has been shown to correlate with increased resistance to both chemotherapy and radiotherapy (19, 20, 25, 26). In line with these *in vitro* studies, in our cohort of clinical samples we identified up-regulation of APE1 in the post-treatment tumor samples, providing further evidence for the evolution of treatment-acquiring resistance mechanisms.

Transcriptional analysis revealed a changing landscape with an immunosuppressive phenotype identified following treatment and disease recurrence. *KLRC1* and *CCL5* have been shown to be involved in compromising anti-tumoral responses with *KLRC1* inhibiting the killing ability of CD8+ T cells (27–32) and *CCL5* recruiting immunosuppressive T regulatory cells into the tumor microenvironment (33, 34). In addition, *CCL5* and *GPNMB* have both been reported to play a role in glioma invasion, migration, and recurrence (35, 36).

We also identified a shift in macrophage polarization with increased *CD163* and *MSR1* identified in post treatment specimens. These M2 phenotypic conditions were also found to enhance migration of glioma cells and markedly enhance migration in post-treatment glioma cells relative to pre-treatment glioma cells. This finding is of particular significance when it is considered that glioma associated macrophages/microglia (GAMs) can comprise up to 30% of the tumor volume (37). This M2-like phenotype is also correlated with glioma grade and aggressiveness (38–40). M2 polarization is currently considered a major mechanism by which cancer cells evade the immune system (41–45). While it is well documented that GAMs have an M2-like phenotype (37, 38, 46, 47), the majority of data did not compare tumors longitudinally to understand whether GAMs begin as M2-like or whether there is a phenotypic shift. Our longitudinal clinical data confirm that GAMs assume a classically activate M1 phenotype early on in tumor growth and as the tumor progresses to a more advanced stage, these GAMs switch to resemble an alternatively activated M2 phenotype (48, 49). This has now been shown to be related to the level of hypoxia within the tumors (50–52).

Overall, these findings highlight the ability of glioblastomas to evade not only the toxic onslaught of therapy but also to evade the immune system, findings that would never have been identified if only the pre-treatment specimen was analyzed. These findings also suggest that a two-pronged approach may be needed, one treatment targeting the treatment resistance mechanism and another targeting the immune suppression. Immune-altering therapies are currently of high interest and are being investigated in many types of cancer. It remains to be determined whether such treatments are also effective in glioblastoma.

## Author contributions

AH, NP, PK, JP, VH, and HW conceived the project. AH, NP, PK, TD, and RI designed and performed the experiments. AH, NP, PK, TD, RI, YZ, JC, and VH analyzed the data. HW and VH advised on the research. AH, NP, HW, and VH wrote the manuscript. All authors reviewed and revised the manuscript.

### Conflict of interest statement

The authors declare that the research was conducted in the absence of any commercial or financial relationships that could be construed as a potential conflict of interest. The reviewer MH and handling Editor declared their shared affiliation.
