# Supplementary material for: Glioblastoma Recurrence Correlates With Increased APE1 and Polarization Toward an Immuno-Suppressive Microenvironment
Source: Front Oncol. 2018 Aug 13;8:314. doi: 10.3389/fonc.2018.00314 (PMC6099184; doi:10.3389/fonc.2018.00314)
Supplement: Supplementary file 1 [file Data_Sheet_1.docx]

*Supplementary Material*

Glioblastoma Recurrence Correlates with Increased APE1 and Polarisation Towards an Immuno-Suppressive Microenvironment

Amanda L. Hudson^*^, Nicole R. Parker, Peter Khong, Jonathon F. Parkinson, Trisha Dwight, Rowan J. Ikin, Ying Zhu, Jason Chen, Helen R. Wheeler and Viive M. Howell.

^*^Corresponding Author

[amanda.hudson@sydney.edu.au](mailto:amanda.hudson@sydney.edu.au)

***Methods***

*Targeted Next Generation Sequencing*

An amplicon library was created using 250 ng of genomic DNA from tumour specimens as per the manufacturer’s instructions (TruSeq Custom Amplicon Library Kit, Illumina, Scoresby, Victoria). A custom oligo panel was designed using Illumina Design Studio software and the UCSC genome browser to improve coverage, avoiding regions of low complexity sequence and CpG rich sequence. Primers were designed against all exonic regions of the six MMR/BER genes of interest (*APEX1, PARP1, MSH2, MSH6, MLH1* and *PMS2*) using a total of 183 amplicons, representing 99% of the coding sequence. The exome library was then pooled and sequenced on the Illumina MiSeq platform as 250-bp paired end reads (MiSeq Reagent Kit v2, 300 cycles) according to the Illumina protocol. Primary analysis of deep sequencing data was performed by the MiSeq Illumina reporter software (using default settings), including alignment of sequence reads to the human genome reference GRCh37 (hg19).

*DNA Variant Identification*

Sequenced reads were mapped to the hg19 reference human genome (Ensembl Gene annotation 2014.01.02) using Avadis NGS v1.6 software (Strand Life Sciences, India). After filtering out low-quality and duplicate reads, single nucleotide variants (SNVs) and short insertions/deletions were identified using pipeline analysis in the Avadis software and default criteria (≥20X coverage, an average base quality ≥20 with a confidence score cutoff greater than 50, ≥10 reads coverage of the specific variant location and ≥2 reads of the variant).

*Cytotoxicity assay*

Assays were performed in triplicate in a final volume of 200 µl. 2 x 10^3^ cells in 100 µl were plated into 96-well microtitre plates and left to adhere for approximately 4 hrs. Serial drug dilutions (100 µl) were then added and plates were incubated for 72 hrs. in standard cell culture conditions. The MTS assay was performed as previously described (Cory, AH et al 1991 PMID: 1867954) to determine cell viability and GraphPad Prism non-linear (curve fit) regression algorithms were used to calculate the drug dose causing 50% growth inhibition (IC50 drug dose).

**SI Table 1: *MGMT* promoter methylation results for determining the assay cut off values.** The cut-off value was determined by averaging the percentage methylation at 4 CpG sites of known unmethylated controls and applying 2 standard deviations. Samples were considered methylated if the average percentage methylation was >13% or unmethylated if the average percentage methylation was <13%. FFPE, formalin fix paraffin embedded.

| **Sample** | **Average % methylation** |
| --- | --- |
| Normal brain FFPE tissue (n=13) | 9.8 |
|  | 1 |
|  | 4.8 |
|  | 4.8 |
|  | 3.5 |
|  | 1.8 |
|  | 2.3 |
|  | 9.5 |
|  | 15.3 |
|  | 11.8 |
|  | 6.5 |
|  | 6 |
|  | 12.5 |
| Normal brain fresh/frozen tissue (n=3) | 4.3 |
|  | 3.8 |
|  | 3.5 |
| Unmethylated control (n=8) | 2 |
|  | 9 |
|  | 5.3 |
|  | 4.8 |
|  | 2.8 |
|  | 2.3 |
|  | 2.5 |
|  | 2 |
|  |  |
| **Mean percentage methylation** | **5.49** |
| **Standard deviation** | **3.85** |
| **Assay cut off Mean + 2 standard deviations** | **13%** |

**SI Table 2: MGMT promoter methylation results for each tumour specimen.**

Average percentage methylation was determined at 4 CpG sites using pyrosequencing. M, methylated MGMT promoter; U, unmethylated MGMT promoter.

| **Case** | **CpG1%** | **CpG2%** | **CpG3%** | **CpG4%** | **Average % methylation** | **Methylation status (M:>13%, U:<13%)** |
| --- | --- | --- | --- | --- | --- | --- |
| 4a | 9 | 11 | 6 | 12 | 9.5 | unmethylated |
| 4b | 7 | 8 | 4 | 8 | 6.75 | unmethylated |
| 5a | 1 | 1 | 3 | 3 | 2 | unmethylated |
| 5b | 1 | 1 | 3 | 3 | 2 | unmethylated |
| 7a | 28 | 15 | 10 | 10 | **15.75** | **unmethylated** |
| 7b | 1 | 1 | 3 | 3 | 2 | unmethylated |
| 10a | 3 | 16 | 5 | 11 | 8.75 | unmethylated |
| 10b | 1 | 1 | 2 | 3 | 1.75 | unmethylated |
| 13a | 2 | 2 | 4 | 4 | 3 | unmethylated |
| 13b | 2 | 2 | 3 | 4 | 2.75 | unmethylated |
| 16a | 2 | 2 | 2 | 2 | 2 | unmethylated |
| 16b | 1 | 1 | 2 | 3 | 1.75 | unmethylated |
| 19a | 0 | 1 | 1 | 1 | 0.75 | unmethylated |
| 19b | 3 | 2 | 2 | 3 | 2.5 | unmethylated |
| 20a | 2 | 2 | 2 | 3 | 2.25 | unmethylated |
| 20b | 1 | 1 | 2 | 3 | 1.75 | unmethylated |
| 23a | 1 | 1 | 2 | 2 | 1.5 | unmethylated |
| 23b | 1 | 2 | 2 | 3 | 2 | unmethylated |
| 25a | 5 | 10 | 29 | 53 | **24.25** | **methylated** |
| 25b | 76 | 31 | 16 | 32 | **38.75** | **methylated** |
| 26a | 8 | 30 | 6 | 11 | **13.75** | **methylated** |
| 26b | 2 | 1 | 2 | 3 | 2 | unmethylated |
| 29a | 1 | 1 | 2 | 2 | 1.5 | unmethylated |
| 29b | 2 | 2 | 4 | 4 | 3 | unmethylated |
| 30a | 15 | 33 | 8 | 46 | **25.5** | **methylated** |
| 30b | 20 | 23 | 6 | 33 | **20.5** | **methylated** |
| 32a | 2 | 2 | 3 | 4 | 2.75 | unmethylated |
| 32b | 3 | 3 | 4 | 4 | 3.5 | unmethylated |
| 33a | 4 | 6 | 5 | 8 | 5.75 | unmethylated |
| 33b | 2 | 3 | 3 | 4 | 3 | unmethylated |
| 35a | 2 | 3 | 3 | 2 | 2.5 | unmethylated |
| 35b | 3 | 4 | 3 | 6 | 4 | unmethylated |
| 38a | 12 | 15 | 5 | 9 | 10.25 | unmethylated |
| 38b | 4 | 4 | 5 | 7 | 5 | unmethylated |
| 40a | 2 | 2 | 6 | 6 | 4 | unmethylated |
| 40b | 0 | 0 | 1 | 2 | 0.75 | unmethylated |
| 42a | 3 | 10 | 3 | 5 | 5.25 | unmethylated |
| 42b | 1 | 1 | 3 | 3 | 2 | unmethylated |

**SI Table 3: TaqMan assays used for gene expression analysis**

|  | **Gene symbol** | **Taqman Probe ID** |
| --- | --- | --- |
| 1 | AKT3 | Hs00987350_m1 |
| 2 | ANXA1 | Hs00167549_m1 |
| 3 | APE1 | Hs00959050_g1 |
| 4 | AQP1 | Hs01028916_m1 |
| 5 | ARG1 | Hs00968979_m1 |
| 6 | ASCL1^@^ | Hs04187546_g1 |
| 6 | ATRX^@^ | Hs00230877_m1 |
| 7 | AURORA A | Hs01582072_m1 |
| 8 | AURORA B | Hs00945858_g1 |
| 9 | CCL2 | Hs00234140_m1 |
| 10 | CCL22 | Hs01574247_m1 |
| 11 | CCL24 | Hs00171082_m1 |
| 12 | CCL5 | Hs00174575_m1 |
| 13 | CCND2 | Hs00153380_m1 |
| 14 | CD163 | Hs00174705_m1 |
| 15 | CD204 | Hs00234007_m1 |
| 16 | CD4 | Hs01058407_m1 |
| 17 | CD44^@^ | Hs01075861_m1 |
| 18 | CD68 | Hs02836816_g1 |
| 19 | CD74 | Hs00269961_m1 |
| 20 | CD80 | Hs00175478_m1 |
| 21 | CDK6 | Hs01026371_m1 |
| 22 | CDKN2A^@^ | Hs00923894_m1 |
| 23 | C/EBPBeta^@^ | Hs00270923_s1 |
| 24 | CTGF | Hs01026927_g1 |
| 25 | CXCR4 | Hs00607978_s1 |
| 26 | DCX^@^ | Hs00167057_m1 |
| 27 | DLL1^@^ | Hs00194509_m1 |
| 28 | DLL3^@^ | Hs01085096_m1 |
| 29 | EGFR^@^ | Hs01076078_m1 |
| 30 | EMP3 | Hs00171319_m1 |
| 32 | FN1^@^ | Hs00365052_m1 |
| 33 | GAPDH | Hs00183533_m1 |
| 34 | GLI2^@^ | Hs01119974_m1 |
| 35 | GPNMB | Hs01095679_m1 |
| 36 | HES1^@^ | Hs00172878_m1 |
| 37 | HES5 | Hs01387463_g1 |
| 38 | HIF1alpha | Hs00153153_m1 |
| 39 | HIF2alpha | Hs01026149_m1 |
| 40 | HMBS | Hs00609293_g1 |
| 41 | IFNgamma | Hs00989291_m1 |
| 42 | IGFBP2 | Hs01040719_m1 |
| 43 | IL6 | Hs00985639_m1 |
| 44 | IL8 | Hs00174103_m1 |
| 45 | ING1 | Hs04186609_m1 |
| 46 | IP10 (CXCL10) | Hs01124251_g1 |
| 47 | IPO8 | Hs00183533_m1 |
| 48 | JAG1^@^ | Hs01070032_m1 |
| 49 | KLRC1 | Hs00970273_g1 |
| 50 | LEF1 | Hs01547250_m1 |
| 51 | LGALS3^*^ | Hs00173587_m1 |
| 52 | LOX | Hs00942480_m1 |
| 53 | LTBP1 | Hs00386448_m1 |
| 54 | MDM4 | Hs00910358_s1 |
| 55 | MET | Hs01565584_m1 |
| 56 | MGMT | Hs01037698_m1 |
| 57 | MLH1 | Hs00179866_m1 |
| 58 | MSH2 | Hs00953527_m1 |
| 59 | MSH6 | Hs00264721_m1 |
| 60 | NBN/NBS1^@^ | Hs01039836_m1 |
| 61 | NCAM1^@^ | Hs00941830_m1 |
| 62 | NCAM2^@^ | Hs00189850_m1 |
| 63 | NDRG1 | Hs00608387_m1 |
| 64 | NF1^@^ | Hs01035108_m1 |
| 65 | NNMT # | Hs00196287_m1 |
| 66 | NOTCH1 | Hs01062014_m1 |
| 67 | NOTCH3 | Hs01128541_m1 |
| 68 | OLIG2^@^ | Hs00300164_s1 |
| 69 | OMG^@^ | Hs03047013_s1 |
| 70 | PARP1 | Hs00242302_m1 |
| 71 | PD1L1 (B7-H1)/CD274 | Hs01125301_m1 |
| 72 | PDGFRA^@^ | Hs00998018_m1 |
| 73 | PDPN | Hs00366766_m1 |
| 74 | PIK3CA | Hs00907957_m1 |
| 75 | PIK3R1 | Hs00933163_m1 |
| 76 | PLP1 | Hs00166914_m1 |
| 77 | PMS2 | Hs00241053_m1 |
| 78 | PTEN | Hs02621230_s1 |
| 79 | PTPN13 | Hs01106214_m1 |
| 80 | PTX3 | Hs00173615_m1 |
| 81 | RTN1^@^ | Hs00382515_m1 |
| 82 | S100A8 | Hs00374264_g1 |
| 83 | S100A9 | Hs00610058_m1 |
| 84 | SDHA | Hs00188166_m1 |
| 85 | SERPINE1^@^ | Hs01126607_g1 |
| 86 | SERPING1 | Hs00163781_m1 |
| 87 | SOX 9^@^ | Hs01001343_g1 |
| 88 | STAT3^@^ | Hs00374280_m1 |
| 89 | STAT5A | Hs00234181_m1 |
| 90 | TBP | Hs00427620_m1 |
| 91 | TGFb1 | Hs00998133_m1 |
| 92 | TIMP1 | Hs00171558_m1 |
| 93 | TNFalpha^@^ | Hs01113624_g1 |
| 94 | TP53 | Hs01034249_m1 |
| 95 | VEGFA^@^ | Hs00900055_m1 |
| 96 | YKL40/CHI3L1^@^ | Hs00609691_m1 |

^@^30-gene panel used for hierarchical clustering; *Data from the AB7900HT platform only

**SI Table 4: Optimised conditions for immunohistochemistry analysis**

| Antibody | Source | Dilution and incubation time | Retrieval conditions | Identified cellular location |
| --- | --- | --- | --- | --- |
| APE1, clone 13B8E5C2 | Novus Biologicals | 1:16,000 for 30 mins | Target Retrieval Solution, pH 9 (Dako) | Nucleus |
| PARP1, clone F-2 | Santa Cruz Biotechnolgy | 1:400 for 30 mins | Target Retrieval Solution, pH 9 (Dako) | Nucleus |
| MSH2, clone FE11 | Calbiochem | 1:50 for 30 mins | Target Retrieval Solution, pH 9 (Dako) | Nucleus |
| MSH6, clone 2D4B5 | Zymed | 1:20 for 60 mins | Universal Decloaker Solution (Biocare Medical) | Nucleus |
| MLH1, clone G168-15 | BD Pharmingen | 1:25 for 30 mins | Target Retrieval Solution, pH 9 (Dako) | Nucleus |
| PMS2, clone A16-4 | BD Pharmingen | 1:50 for 30 mins | Target Retrieval Solution, pH 9 (Dako) | Nucleus |

SI Figure 1

SI Figure 1: Cell viability of pre- and post- treatment glioma cells following temozolomide administration as determined by MTS assays. Points show the mean and standard derivation of triplicate wells. The drug dose causing 50% growth inhibition (IC50 drug dose) was determined and the p-value was calculated by comparing the IC50 values from four independent experiments.

SI Figure 2:

SI Figure 2. Gene expression analysis of BER and MMR pathways genes. The gene expression of Base Excision Repair (BER) genes (APEX1 and PARP1) and Mismatch Repair (MMR) genes (MSH6, MSH2, MLH1 and PMS2) was examined in tumor biopsies pre- and post-adjuvant therapy (indicated by solid circle and open squares, respectively) relative to normal brain tissue (indicated by the broken line). Results were generated using Taqman assays and TBP as the reference for normalization. A 2-fold change in gene expression relative to normal brain is indicated by the solid grey line. Purple symbols represent specimens predicted to contain deleterious gene specific DNA variants.

SI Figure 3

SI Figure 3: Targeted next-generation exome sequencing results for BER (APEX and PARP1) and MMR (MSH2, MSH6, MLH1 and PMS2) pathways genes. The Illumina MiSeq platform was used together with Avadis NGS software for analysis. The total number of sequence variants identified for all the genes are grouped together. All reads had an average base quality greater than 20, a confidence score cutoff greater than 50 and variant reads greater than 2. The depth of coverage ranged from 10 – 3500x. No significant difference was identified between the groups (ns).

SI Figure 4

SI Figure 4: Two gene scatter plot for selected genes significantly increased following treatment and recurrence of disease. Data was retrieved from Project Betastatis using the REMBRANDT repository (n=425). Pearson correlation coefficients were calculated using GraphPad prism with an r value of >0.5 indicating a moderate and >0.6 indicating a strong correlation.
